# UBE2S promotes malignant properties via VHL/HIF‐1α and VHL/JAK2/STAT3 signaling pathways and decreases sensitivity to sorafenib in hepatocellular carcinoma

**DOI:** 10.1002/cam4.6431

**Published:** 2023-08-10

**Authors:** Junyi Wu, Xiangjie Xu, Shasha Wu, Weiwei Shi, Guang Zhang, Yin Cao, Zhongxia Wang, Junhua Wu, Chunping Jiang

**Affiliations:** ^1^ Jinan Microecological Biomedicine Shandong Laboratory Shounuo City Light West Block Jinan Shandong China; ^2^ Shengli Clinical Medical College of Fujian Medical University Fuzhou Fujian China; ^3^ Nanjing Drum Tower Hospital Clinical College of Traditional Chinese and Western Medicine Nanjing University of Chinese Medicine Nanjing Jiangsu China; ^4^ Department of Clinical Medicine and Rehabilitation Jiangsu College of Nursing Huai'an Jiangsu China; ^5^ State Key Laboratory of Pharmaceutical Biotechnology, National Institute of Healthcare Data Science at Nanjing University, Jiangsu Key Laboratory of Molecular Medicine Medical School of Nanjing University, Nanjing University Nanjing Jiangsu China

**Keywords:** malignant properties, sorafenib, UBE2S, VHL

## Abstract

**Background:**

Ubiquitin‐conjugating enzyme E2S (UBE2S), an E2 enzyme, is associated with the development of various tumors and exerts oncogenic activities. UBE2S is overexpressed in tumors, including hepatocellular carcinoma (HCC). However, the key molecular mechanisms of UBE2S in HCC still need additional research. The aim of this study was to explore the role of UBE2S in HCC.

**Methods:**

The expression levels of UBE2S in HCC tissues and cells were detected by western blot analysis, quantitative real‐time polymerase chain reaction analysis (qRT–PCR), and immunohistochemistry (IHC). A 3‐(4,5‐dimethylthiazol‐2‐yl)‐2,5‐diphenyl‐2H‐tetrazolium bromide (MTT) assay, wound healing assay, colony formation assay transwell assay, and animal models were used to detect the proliferation and migration ability of HCC cells. Western blot analysis, qRT–PCR, immunofluorescence, small‐interfering RNA (siRNA), and plasmid transfection and coimmunoprecipitation (Co‐IP) assays were performed to detect the interaction among UBE2S, von Hippel–Lindau (VHL), hypoxia‐inducible factor 1‐alpha (HIF‐1α), Janus kinase‐2 (JAK2), and signal transducer and activator of transcription 3 (STAT3).

**Results:**

In this study, we found that high UBE2S expression was associated with poor prognosis in HCC patients. In addition, UBE2S expression was upregulated in HCC tissues and cell lines. Knockdown of UBE2S inhibited the proliferation and migration of HCC cells in vitro and in vivo by directly interacting with VHL to downregulate the HIF‐1α and JAK2/STAT3 signaling pathways. Accordingly, overexpression of UBE2S significantly enhanced the proliferation and migration of HCC cells in vitro via VHL to upregulate HIF‐1α and JAK2/STAT3 signaling pathways. Furthermore, we found that downregulation of UBE2S expression enhanced the sensitivity of HCC cells to sorafenib in vivo and in vitro.

**Conclusion:**

UBE2S enhances malignant properties via the VHL/HIF‐1α and VHL/JAK2/STAT3 signaling pathways and reduces sensitivity to sorafenib in HCC. The findings of this study may open a new approach for HCC diagnosis and provide a potential option for the treatment of HCC.

## 1 INTRODUCTION

Hepatocellular carcinoma (HCC) is the most diagnosed liver cancer and is the third leading cause of cancer‐related death worldwide.^1^ According to epidemiological statistics, approximately 800,000 new cases occur worldwide every year.^2^ Although many studies have focused on diagnostic strategies and treatment approaches for HCC,^3^, ^4^ the 5‐year survival rate has remained below 12%.^5^ At present, surgical resection remains the most effective treatment for HCC. However, many HCC patients are diagnosed in an advanced stage and have lost the chance for surgical resection. In addition, the potential of invasion and metastasis in HCC is the main factor causing a poor prognosis.^3^, ^4^ These factors cause HCC to be one of the most malignant tumors. Before August 2018, the targeted systemic treatment that was available for advanced HCC patients was mainly the tyrosine kinase inhibitor (TKI) sorafenib (first‐line), which can improve prognosis of HCC patients. After August 2018, lenvatinib and atezolizumab plus bevacizumab also became the first‐line systemic molecular therapy effective in HCC. However, sorafenib is effective only in certain HCC patients, and its side effects are serious, such as gastrointestinal bleeding, respiratory bleeding, neutrocytopenia, and lymphocytopenia.^6^ More seriously for some advanced HCC, sorafenib would promotes proliferation and metastasis.^7^ In addition, after sorafenib resistance develops, only regorafenib, cabozantinib, and ramucirumab yield a positive response as second‐line treatments for HCC.^8^ In sum, it is still a great challenge to estimate HCC progression and responsiveness to TKI therapies due to a lack of molecular biomarkers. Therefore, it is necessary to study the key molecular mechanisms underlying HCC pathogenesis, which could uncover biomarkers for early diagnosis, prognosis, or targeted treatment.

The ubiquitin‐conjugating enzyme E2S (UBE2S), an E2 enzyme, is a key degradation enzyme that participates in the ubiquitination of proteins.^9^ Ubiquitin binds to target proteins that are then degraded via the ubiquitin–proteasome system in the process of ubiquitination, which contains three types of enzymes: E1 (activating enzyme), E2 (binding enzyme), and E3 (ubiquitin ligase).^10^ UBE2S, a K11 linkage‐specific E2, can prolong K11‐linked polyubiquitin chains on substrates through cooperation with Ube2c/d or anaphase‐promoting complex/cyclosome (APC/C).^11^, ^12^, ^13^ Moreover, UBE2S can regulate differentiation of Sox2‐mediated embryonic stem cell and associate with Ku70 for DNA repair.^14^ Recently, many studies have indicated that UBE2S is overexpressed in multiple human primary cancers and is involved in tumorigenesis.^15^, ^16^, ^17^ Overexpression of UBE2S in tumors could regulate the cell cycle and increase cell proliferation, metastasis, and invasiveness.^16^, ^17^, ^18^, ^19^ In addition, the high expression of UBE2S in glioblastoma multiforme induces resistance to chemotherapy.^20^

Von Hippel–Lindau (VHL) is a tumor suppressor gene and the substrate‐conferring component of the E3 ubiquitin ligase complex.^15^ Mutations in VHL cause a hereditary cancer syndrome, and loss of VHL is associated with tumor progression and angiogenesis.^15^ Studies have found that UBE2S can specifically ubiquitinate and degrade VHL via the 26S proteasome to stabilize hypoxia‐inducible factor (HIF) and eventually promote tumor progression.^15^, ^21^, ^22^ Furthermore, Kasamatsu et al. found that UBE2S enhances oral squamous cell carcinoma (OSCC) proliferation by promoting p21 degradation.^16^ In addition, UBE2S mediates colorectal cancer development by stabilizing β‐catenin via K11‐linked polyubiquitination.^23^ Interestingly, research by Pan et al. revealed that in HCC, high expression of UBE2S promotes the ubiquitination of p53, which can increase cell proliferation and migration.^17^ In addition, UBE2S could binding interact with tripartite motif protein 28 (TRIM28) in the nucleus and accelerates the cell cycle by ubiquitination of p27^19^ and enhances the ubiquitination of p53 to promote HCC development.^17^ However, the key molecular mechanisms of the oncogenic activities of UBE2S in HCC and the role of UBE2S in the sensitivity of HCC to sorafenib are far from well understood. Therefore, a more in‐depth study of the molecular mechanism underlying the effects of UBE2S in HCC and the role of UBE2S in sorafenib sensitivity in HCC is extremely meaningful. This study was designed to investigate the role of UBE2S in the malignant properties of HCC and HCC cell sensitivity to sorafenib in vivo and in vitro and to explore the underlying molecular mechanisms.

## 2 MATERIALS AND METHODS

### 2.1 Clinical specimens

All human primary HCCs, paired peritumoral liver tissues and nontumor liver tissues were collected from the Department of Hepatobiliary Surgery, the Affiliated Drum Tower Hospital of Nanjing University Medical School. Among them, 49 pairs of tumor and paired peritumoral liver tissues were collected to detect the mRNA levels of UBE2S, and 8 of 49 were collected to detect protein levels of UBE2S. None of the patients received any other antitumor treatment before surgery such as chemotherapy, radiotherapy, targeted therapy. The use of human tumor samples was approved by the Ethics Committee of the Affiliated Drum Tower Hospital of Nanjing University Medical School.

### 2.2 Cell lines and cell culture

The LO_2_ (human embryonic liver) cell line and the Huh7 (human HCC) cell line were purchased from the Cell Bank of Xiangya Central Laboratory, Central South University. Three liver cancer cell lines, HCCLM3, MHCC‐97 L, and MHCC‐97H, were gifts from the Liver Cancer Institute, Zhongshan Hospital, Fudan University. SMMC‐7721, HepG2, Bel‐7402, Hep3B, PLC, and Li‐7 cell lines were obtained from the cell bank of the Chinese Academy of Sciences. HCCLM3, MHCC‐97 L and MHCC‐97H, HepG2, Hep3B, SMMC‐7721, PLC, Huh7, and Li‐7 cells were cultured in complete DMEM (Wisent, Inc.). LO2 and Bel‐7402 cells were cultured in complete RPMI‐1640 medium (Wisent, Inc.). All cells were supplemented with 10% fetal bovine serum (Wisent, Inc.) at 37°C in a humidified incubator with 5% CO_2_.

### 2.3 Western blot analysis

Cells were lysed with NP40 solution (Beyotime Biotechnology) containing 1% protease inhibitors (Thermo Fisher Scientific). A BCA protein assay kit (Thermo Fisher Scientific) was used to quantify proteins. Subsequently, the proteins were boiled in loading buffer, fractionated by sodium dodecyl sulfate–polyacrylamide gel electrophoresis (SDS–PAGE) and then transferred onto polyvinylidene difluoride (PVDF) membranes. After that, they were stained with primary antibodies overnight at 4°C, and the protein bands were incubated with horseradish peroxidase (HRP)‐conjugated secondary antibody and detected using enhanced chemiluminescence (EMD Millipore, Billerica, MA, USA) with a Tanon 5200 Chemiluminescent Imaging System (Tanon Science and Technology Co., Ltd.). Antibodies against UBE2S, cyclin B, P‐CDC20, CDC20, E‐cadherin, N‐cadherin, vimentin, Snail, JAK2, p‐JAK2, STAT3, p‐STAT3, and p21 were purchased from Cell Signaling Technology. Antibodies against VHL and HIF‐1α were purchased from Proteintech (Proteintech Group, Inc.). Antibodies against cyclin A and cyclin E2 were purchased from Santa Cruz Biotechnology (Santa Cruz Biotechnology). The dilution of the primary antibodies was 1:1000. The dilution of anti‐GAPDH (Bioworld Technology, Inc.) was 1:10,000.

### 2.4 Quantitative real‐time polymerase chain reaction analysis (qRT–PCR)

The primer sequences of human UBE2S and GAPDH were as follows: UBE2S: (5′‐3′) CGACACGTACTGCTGACCAT, (5′‐3′) GCCGCATACTCCTCGTAGTT, and GAPDH: (5′‐3′) CCATGTTCGTCATGGGTGTGAACCA, (5′‐3′) GCCAGTAGAGGCAGGGATGATGTTC. Total RNA was extracted through TRIzol® reagent (Takara Bio, Inc.) and converted to cDNA with PrimeScript RT Master Mix (Takara Bio, Inc.). SYBR Premix Ex Taq II (Takara Bio, Inc.) was used to detect the relative mRNA expression levels, which was calculated by the 2^−ΔΔCq^ method with GAPDH.

### 2.5 Immunohistochemistry (IHC)

Protein levels were detected by IHC as previously described.^17^ Human HCC specimens and HCCLM3 tumor tissues were collected from nude mice and fixed in 10% formalin. The tumor tissues were dehydrated in an ascending ethanol series, then cleared with xylene and embedded in paraffin. They were incubated with the primary antibody at 4°C overnight, after tumor tissue sections were prepared. The tumor tissue sections were washed with PBST three times. Then, they were inoculated with HRP‐conjugated secondary antibody and developed using the 3,3′‐diaminobenzidine (DAB) substrate. The tissue sections were further counterstained with haematoxylin. Stained sections were examined and photographed via an Olympus BX51 microscope. The percentage of positively stained cells was scored as 0: 0%; 1: 1%–25%; 2: 26%–50%; 3: 51%–75%; 4: 76%–100%. The intensity was scored as 0: negative staining; 1: weak staining; 2: moderate staining; 3: strong staining. The results were performed manually by two experienced pathologists. The product of the staining intensity score and the percentage score was scored as 0: 0, 1–4: +, 5–8: ++, 9–12: +++. According to the UBE2S levels in HCC tissue detected by IHC, 103 patients with HCC were divided into two groups: the high UBE2S expression group (score: ++, +++, *n* = 57) and the low UBE2S expression group (score: 0, +, *n* = 46). The primary antibodies used for IHC were anti‐UBE2S (ab197945, 1:200, Abcam), anti‐p‐JAK2 (ab32101, 1:200, Abcam), anti‐VHL (16538‐1‐AP, 1:400, Proteintech), anti‐HIF‐1α (20960‐1‐AP, 1:200, Proteintech), anti‐p21 (2947, 1:200, Cell Signaling Technology), anti‐Ki67 (9027, 1:400, Cell Signaling Technology), and anti‐p‐STAT3 (9145, 1:100, Cell Signaling Technology). The secondary antibody for IHC was Super Sensitive TM IHC Detection System Kit (BD5001, Bioworld). The integral optical density (IOD) from IHC staining of anti‐VHL, anti‐HIF‐1α, anti‐p21, anti‐Ki67, anti‐p‐STAT3, and anti‐p‐JAK2 was calculated using Image‐Pro Plus software, and three samples were used for statistics.

### 2.6 Cell proliferation assay

Cells were plated into 96‐well plates at the same concentration of 5 × 10^3^ cells/well for equal amounts of cell seeding. After culturing for 48, 72, and 96 h, MTT solution (5 mg/mL, 20 μL/well) was added to each well for 4 h. Then, 150 μL of DMSO was added to each well to dissolve the insoluble crystals. A spectrophotometer was used to measure the absorbance at 570 nm. Each assay was carried out at least three times.

### 2.7 Colony formation assay

Cells were seeded into 6‐well plates (500 cells/well) for 2 weeks. After colony formation, the medium was discarded. After washing with PBS three times, the cells were fixed with methanol and stained with crystal violet for 15 min. The cell colonies were photographed and counted.

### 2.8 Wound healing assay

Wound healing culture inserts (Ibidi) were used to count migration capacity. First, the cells were seeded into each well of a culture insert (40,000 cells/well). After incubation overnight, the culture insert was removed and were carefully washed with PBS. Then, they were cultured with DMEM or RPMI‐1640 medium without FBS. The cells migrated into the cell‐free area, and the migration was monitored and photographed with an Olympus BX51 microscope at 24, 48, and 72 h. ImageJ software was used to calculate the wound closure rate. All experiments were repeated three times.

### 2.9 Transwell assay

Transwell inserts (Merck Millipore) were used to detect the migration capacity. The upper chamber with or without Matrigel (354230) coating (BD) for 30 min at 37°C for invasion and migration tests, respectively, was seeded with 20,000 HCC cells resuspended in DMEM without FBS. Then, the lower chamber was added with 0.8 mL DMEM supplemented with 10% FBS. At the end of the experiment, the medium was removed. The upper chamber membrane was fixed with methanol. The cells on the upper side were removed via a cotton swab. Then, the transwell inserts were stained with crystal violet. The cells on the opposite side of the membrane were photographed and counted in six random fields with an Olympus BX51 microscope.

### 2.10 Immunofluorescence

Round coverslips were placed into 24‐well plates, and cells were seeded onto the coverslips. After 24 h, the cells were fixed with 4% formaldehyde and permeabilized for 30 min. Then, they were washed with PBS three times, and 500 μL of 0.2% Triton X‐100 was added to the 24‐well plate for 15 min. After the cells were blocked with 1% BSA for 1 h, they were incubated with primary antibody overnight at 4°C. Then, they were washed with PBST three times, and the cells were incubated with FITC‐conjugated goat anti‐rabbit IgG (Zenon® Alexa Fluor® 546 Rabbit IgG Labelling Kit, Thermo Fisher Scientific) for 2 h. After washing with PBS three times, the cells were incubated with DAPI (Thermo Fisher Scientific) for 15 min before being mounted with ProLong Gold Antifade Reagents. Finally, the cells were photographed through a fluorescence confocal microscope (500 IX71, Olympus). The primary antibodies used for immunofluorescence were anti‐VHL (16538‐1‐AP, 1:400, Proteintech), anti‐HIF‐1α (20960‐1‐AP, 1:200, Proteintech), anti‐vimentin (5741, 1:200, Cell Signaling Technology), and anti‐E‐cadherin (14472, 1:400, Cell Signaling Technology).

### 2.11 Small interfering RNA (siRNA) and plasmid transfection

The sequences of human UBE2S siRNA (siUBE2S) and VHL siRNA (siVHL) were as follows: siUBE2S sense (5′‐3′): GACACGUACUGCUGACCAUTT and siVHL sense (5′‐3′): CCAAUGGAUUCAUGGAGUA, which were purchased from GenePharma. Lipofectamine® RNAiMAX (Thermo Fisher Scientific) was used to transfect cells according to the manufacturer's instructions.

The pcDNA3.1‐UBE2S, ‐VHL, and ‐p21 plasmids were synthesized by GeneChem (Shanghai GeneChem Co., Ltd.). The pcDNA3.1 empty vector was used as a negative control. The plasmids were delivered into cells via Lipofectamine 3000 transfection reagent (Thermo Fisher Scientific). The cells were transfected at a density of 80%–90%.

### 2.12 Cell cycle analysis

Cells (5 × 10^5^) were seeded into 6‐well plates. The cells were harvested and fixed with cold 70% ethanol overnight at −20°C after 24 h. Then, after washing with PBS, they were stained in a solution with propidium iodide (PI) (0.5 mg/mL) and RNase A (10 mg/mL). Flow cytometry (FACSCalibur, BD) was used to confirm the cell cycle qualitatively.

### 2.13 Apoptosis assay

Cells (1 × 10^6^) were seeded into 6‐well plates. After 24 h, the cells were collected including apoptotic, dead, and adherent cells. Then, the cells were washed with cold PBS and resuspended in annexin V‐FITC binding buffer. Apoptosis was detected by flow cytometry (FACSCalibur, BD) after incubation with annexin V‐FITC/PI. A TUNEL Apoptosis Assay Kit (Roche) was used for TUNEL staining according to the manufacturer's instructions.

### 2.14 HCC cells with stable UBE2S knockdown and overexpression

Lentivirus vector targeting UBE2S was synthesized by Shanghai GeneChem Company, Ltd. (GeneChem). Transfection efficiency was detected via GFP in the lentiviral vectors. Cells were transfected with constructs of the UBE2S firefly luciferase reporter for 48 h in order to stably knockdown or overexpress corresponding genes. After approximately 80% of the cells were transfected, puromycin (1 μg/mL) was added to the cells for approximately 2 weeks to generate stably transfected cells.

### 2.15 Coimmunoprecipitation (co‐IP) assay

For Co‐IP, the cells were lysed with NP40 (Beyotime Biotechnology) solution containing 1% protease inhibitors. Then, the cell lysates were incubated with antibodies against UBE2S, VHL, or IgG at 4°C overnight, and then incubated with protein A + G Sepharose beads (Beyotime Biotechnology) for another 1 h. Eluted proteins were resolved via 12% SDS–PAGE, and detected by western blotting with antibodies of UBE2S, VHL, or IgG.

### 2.16 Animal models

All animal experimentation were approved by the Animal Care Committee of Nanjing University in accordance with the guidelines of the Institutional Animal Care and Use Committee. The subcutaneous xenograft model in nude mice was performed as previously described.^24^, ^25^ HCCLM3 cells (control‐shRNA group and UBE2S‐shRNA group) and Bel‐7402 cells (control group and UBE2S overexpression group) were resuspended with PBS and then injected into the right axillary region of 4‐ to 5‐week‐old male nude mice (2 × 10^6^ cells in 200 μL of PBS per mouse). The longest (L) and shortest (W) tumor diameters and mouse body weights were measured. Tumor volume (mm^3^) = 1/2 × L × W2. All mice were sacrificed 25 days after the subcutaneous HCCLM3 cell injection and 16 days after the subcutaneous Bel‐7402 cell injection, and the tumor weights were measured. The tumors were fixed in 10% phosphate‐buffered formalin for IHC staining. For sorafenib treatment, there were three groups (control‐shRNA, control‐shRNA+sorafenib, and UBE2S‐shRNA+sorafenib). Seven days after the subcutaneous cell injection, the control‐shRNA+sorafenib and UBE2S‐shRNA+sorafenib groups were intraperitoneally injected with sorafenib (50 mg/kg) daily for 4 weeks. 0.4% dimethyl sulfoxide (DMSO) in PBS was used as negative control. The volumes were measured every 7 days, and after 35 days, all mice were sacrificed.

Orthotopic xenograft tumor models were created as previously described.^26^, ^27^ The HCCLM3 tumor tissues, which were obtained from the subcutaneously transplanted tumor model in the nude mice, were cut into small pieces (1 × 1 × 1 mm). Then, they were implanted into the livers of nude mice to develop the transplanted tumor model (control‐shRNA group and UBE2S‐shRNA group). All mice were sacrificed 8 weeks after the orthotopic xenograft tumor model was formed, and the tumor weight and volume were measured. The lungs and livers were harvested for histopathological examination. For sorafenib treatment, there were four groups (control‐shRNA, UBE2S‐shRNA, control‐shRNA+sorafenib, and UBE2S‐shRNA+sorafenib). Seven days after the transplanted tumor model developed, the control‐shRNA+sorafenib and UBE2S‐shRNA+sorafenib groups were intraperitoneally injected with sorafenib (50 mg/kg) daily for 25 days, and 0.4% dimethyl sulfoxide (DMSO) in PBS was intraperitoneally injected to the control group. The volumes and weights of the tumors were measured. All mice were sacrificed through cervical dislocation at the indicated time points. Inhalation anaesthesia with isoflurane was used in all experimental procedures using animals.

### 2.17 Haematoxylin and eosin (H&E) staining

Lungs obtained from the subcutaneously transplanted tumor model were fixed in 4% paraformaldehyde. The tissue sections were embedded in paraffin and stained with H&E as previously described.^28^ The metastatic nodules in the lungs were photographed and counted using an Olympus BX51 microscope.

### 2.18 Bioinformatics analysis

The RNA‐seq and clinical data of different cancer categories were obtained from the TCGA database (https://gdc.cancer.gov, up to January 28, 2016). The microarray datasets (GSE14520, GSE54236) were downloaded from GEO (http://www.ncbi.nlm.nih.gov/geo/). The level of UBE2S was analyzed by a log2‐fold‐change. Average gene expression was calculated for all involved genes for one sample to analyse the expression level of the pathway gene. The average value represents the sample's average gene expression of the pathway as previously reported.

### 2.19 Statistical analysis

All experimental data were presented as the mean ± standard deviation and analyzed using Student's *t*‐test. Patient survival curves were generated via the Kaplan–Meier method. The correlation between different protein levels was determined by Spearman's rank analysis. When *p* < 0.05, the data were considered significantly different.

## 3 RESULTS

### 3.1 UBE2S was overexpressed in human HCCs and associated with poor prognosis

**FIGURE 1:**
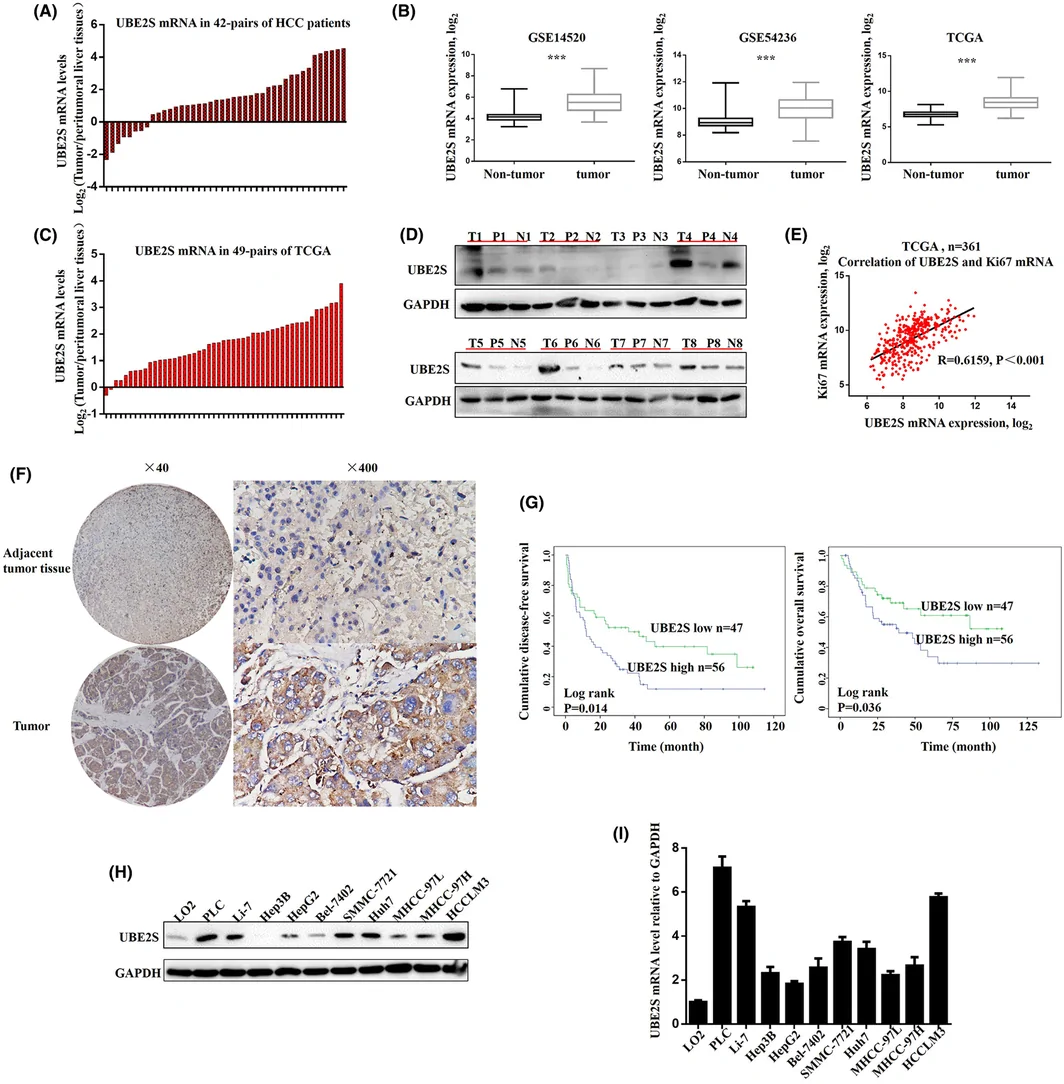
UBE2S is upregulated in human HCC and is associated with poor prognosis. (A) UBE2S expression in 42 pairs of HCC tumor tissues and matched peritumoral tissues was analyzed by RT–PCR. (B) The mRNA levels of UBE2S in HCC tumor tissues and nontumor liver tissues from the TCGA database (373 HCC tumor tissues and 50 nontumor liver tissues) and GEO database (GSE14520 (247 HCC tumor tissues and 239 nontumor liver tissues) and GSE54236 (76 HCC tumor tissues and 76 nontumor liver tissues)) were analyzed. (C) The mRNA level of UBE2S in 49 pairs of HCC tumor tissues and matched peritumoral tissues from the TCGA database was analyzed. (D) The protein level of UBE2S in 8 sets of HCC tumor tissues and nontumor tissues was analyzed by western blotting (N: normal; P: peritumoral; T: tumoral). (E) Spearman correlation analysis between the UBE2S and Ki‐67 mRNA expression levels in the TCGA database. (F) Representative immunohistochemical images showed that the UBE2S protein was more highly expressed in HCC tumor tissues than in peritumoral tissues by IHC staining analyses. Magnification, ×40, ×400. (G) Kaplan–Meier plot of disease‐free survival and overall survival of patients with HCC based on UBE2S levels. (H, I) The protein and mRNA expression of UBE2S in human HCC cell lines and L02 cells were measured by western blotting and qRT–PCR, respectively. UBE2S mRNA expression was represented as the fold change compared to LO2 cells. Data are shown as the mean ± SD. *p < 0.05; **p < 0.01; ***p < 0.001.

The mRNA levels of UBE2S in 42 pairs of HCC tissues and matched peritumoral tissues were first detected by RT–PCR. The results indicated that upregulation of UBE2S mRNA, log2 (the concentration of UBE2S mRNA in tumor tissue/that in matched peritumoral tissue) > 1, was detected in 29 of 42 (69%) HCCs (Figure 1A). The upregulation of UBE2S mRNA was also confirmed by analysis of TCGA data, GSE14520 and GSE54236 in HCC (Figure 1B). In addition, we also analyzed 49 pairs of HCC tumor tissues and their matched peritumoral tissues from the TCGA database (Figure 1C), which also had similar results to those illustrated in Figure 1A. Moreover, we randomly selected eight pairs of tumors to detect the protein level of UBE2S in tumor tissues, measured by western blotting. The results indicated that the protein levels of UBE2S were significantly upregulated in HCC tissues (Figure 1D). Spearman correlation analysis showed that there was a positive correlation between the UBE2S and Ki‐67 mRNA expression levels, which was used as a proliferation marker for human tumor cells (Figure 1E). It indicated that upregulation of UBE2S may promote cell proliferation in HCC. Then, we measured the protein levels of UBE2S in 103 pairs of HCC tissues by IHC. The protein levels of UBE2S were higher in HCC tumor tissues than that in peritumoral liver tissues (Figure 1F). In addition, the results showed that HCC patients with high UBE2S expression had shorter overall survival and disease‐free survival (Figure 1G). We also analyzed the associations between clinicopathological parameters and UBE2S expression levels. The results showed that the high‐expression group had higher clinical stages (*p* = 0.021, Table S1) and larger tumor size (*p* = 0.032, Table S1), compared with the low UBE2S expression group. However, there was no significant difference in other clinical features between the two groups, including sex, age, tumor number, HBV and AFP level. Moreover, we further measured the mRNA and protein levels of UBE2S in LO_2_ and HCC cell lines. We found that the UBE2S levels in the HCC cell lines were significantly higher than those in LO2 cells (Figure 1H,I). In conclusion, these results showed that UBE2S is overexpressed in HCC, which suggested that UBE2S is associated with HCC development. UBE2S may be a potential prognostic biomarker for HCC. These results were consistent with previous studies.^17^

### 3.2 UBE2S promoted HCC cell proliferation, migration, and invasion in vitro

**FIGURE 2:**
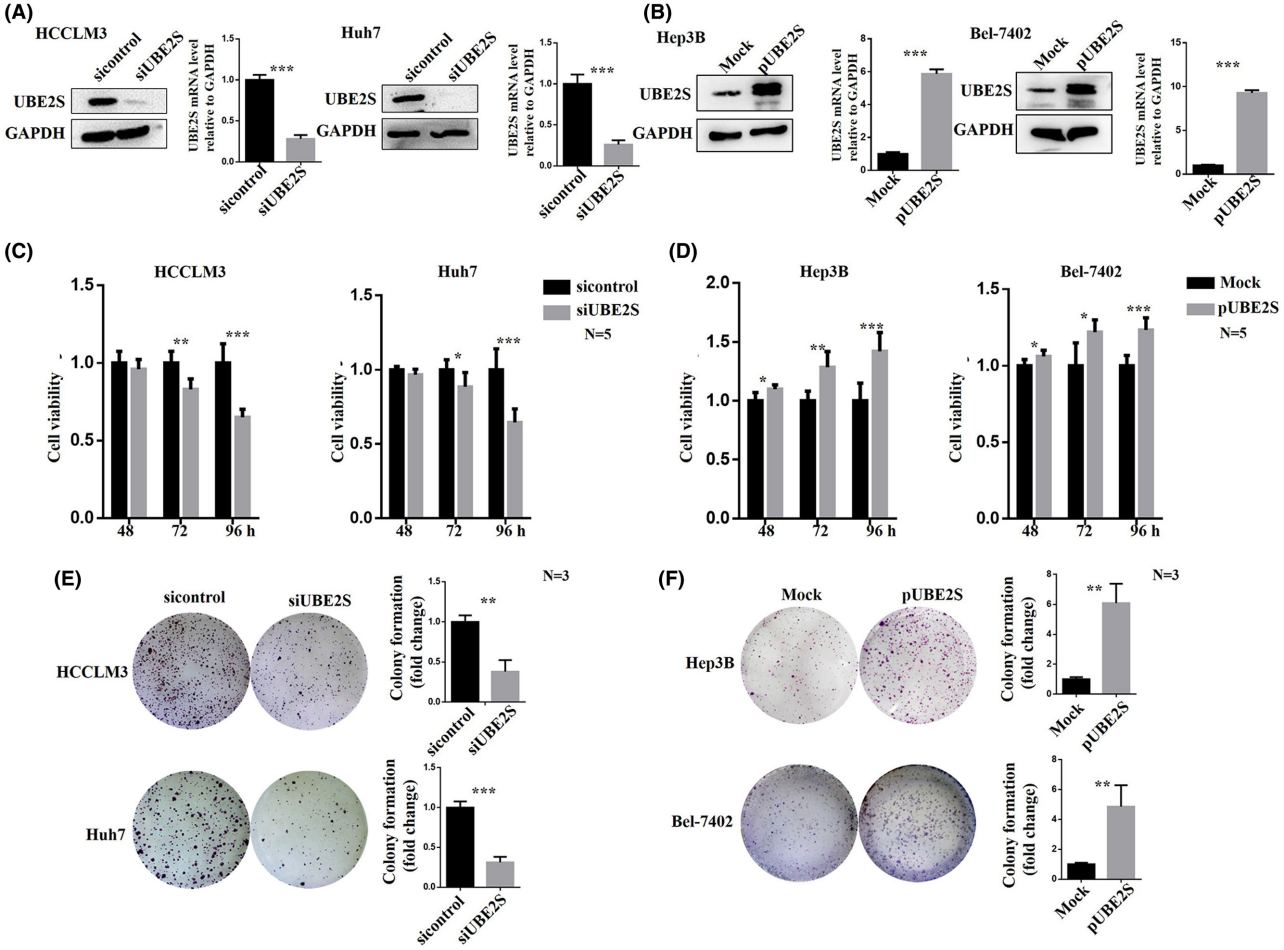
UBE2S promotes HCC cell proliferation in vitro. (A, B) The interference and overexpression efficiency of UBE2S in the HCC cell lines were measured by western blotting and qRT–PCR. (C, D) The effects of UBE2S on HCC cell proliferation were detected at different time points through MTT assays after downregulation and upregulation of UBE2S. (E, F) Representative images and quantification of cell clones in HCC cell lines after downregulation and upregulation of UBE2S. The data are presented as the mean ± SD. NS, no significance; *p < 0.05; **p < 0.01; ***p < 0.001.

**FIGURE 3:**
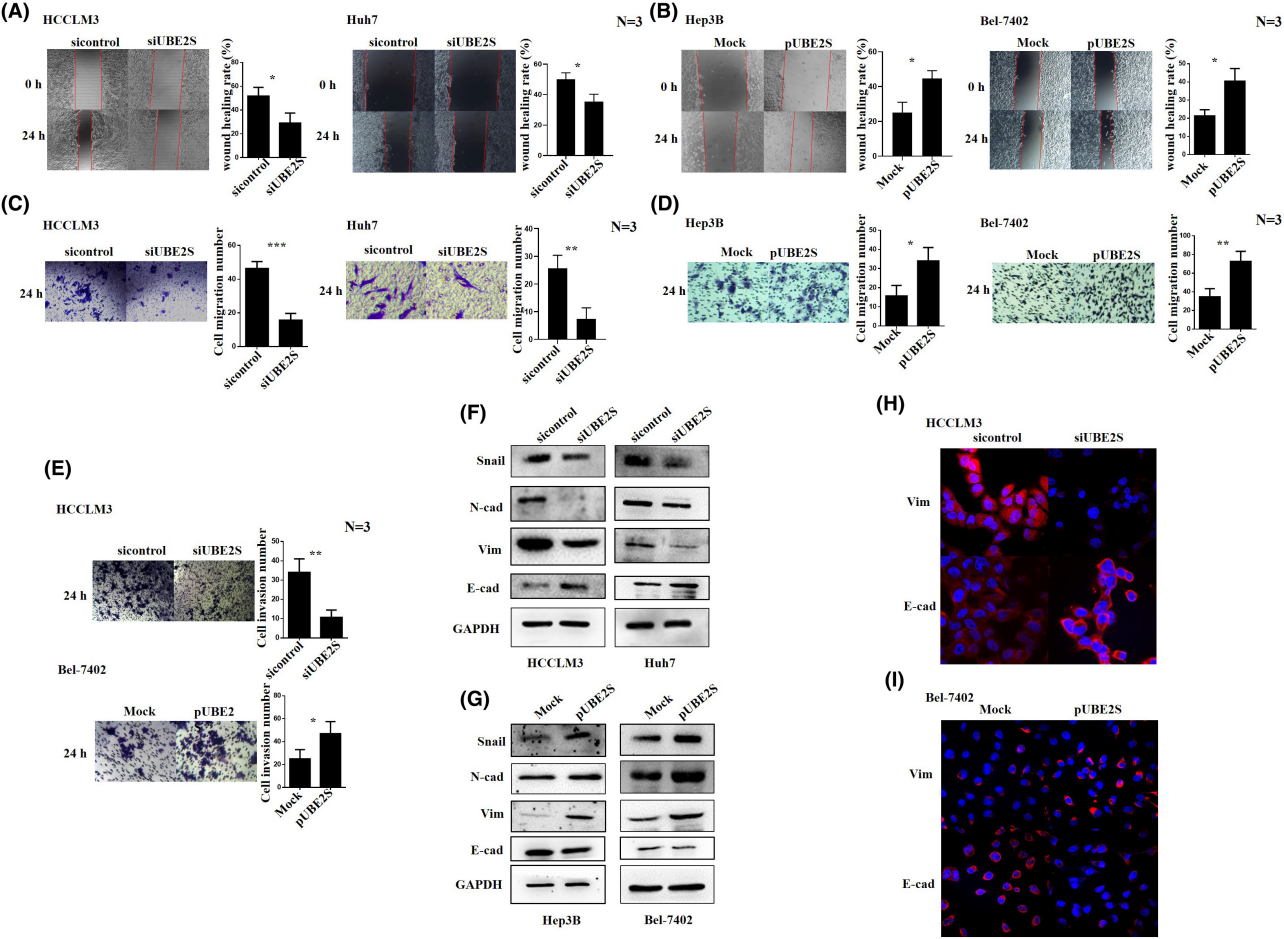
UBE2S promotes HCC cell migration and invasion in vitro. (A, B) The migratory capacity of HCC cell lines after downregulation and upregulation of UBE2S was measured by a wound healing assay. Quantitation of the cell wound healing rate is shown in the right panel. (C, D) Transwell assays were used to measure the migration potential of HCC cell lines after downregulation and upregulation of UBE2S. Quantitation of cell migration is shown in the right panel. (E) Transwell plates coated with Matrigel were used to measure the invasion potential of HCC cell lines after down‐ and upregulation of UBE2S. Quantitation of cell migration is shown in the right panel. (F, G) The effects of upregulation and downregulation of UBE2S on E‐cadherin, N‐cadherin, vimentin, and Snail. (H, I) The effects of upregulation and downregulation of UBE2S on E‐cadherin, and vimentin were tested by immunofluorescence assays. Each bar represents the mean ± SD. *p < 0.05; **p < 0.01; ***p < 0.001.

To further investigate the role of UBE2S in HCC cell proliferation, migration, and invasion, we downregulated the expression level of UBE2S through siRNA transfection (siUBE2S and sicontrol) and overexpressed UBE2S via plasmid transfection (pUBE2S and MOCK). The choice of those specific cells to perform UBE2S downregulation or upregulation depended on the UBE2S levels in the HCC cell lines (Figure 1H). HCCLM3 and Huh7 cells with high expression levels of UBE2S were used to downregulate the expression of UBE2S, while Hep3B and Bel‐7402 cells with low expression levels of UBE2S were used to upregulate the expression of UBE2S. First, we detected the interference and overexpression efficiency of UBE2S in HCC cell lines through western blotting and RT–PCR (Figure 2A,B). The reason for the two UBE2S western blot bands may be that UBE2S degradation is stimulated in HCC cells after UBE2S overexpression (partial degradation), and the protein that has not been completely degraded could still bind to the antibody. Then, the effects of UBE2S on cell proliferation were analyzed by an MTT assay. The results indicated that at 48 h, no significant difference in cell proliferation was observed between the siUBE2S and sicontrol groups. However, the cell proliferation rate was significantly inhibited after downregulation of UBE2S at 72 and 96 h. In addition, overexpression of UBE2S in Hep3B and Bel‐7402 cells induced faster cell growth than that in control cells at 48, 72, and 96 h. Over time, the effect of UBE2S on cell proliferation was increased. The results showed that the cell proliferation rate was significantly inhibited after downregulation of UBE2S (Figure 2C) and increased after overexpression of UBE2S (Figure 2D). A colony formation assay also indicated that after downregulation of UBE2S, the number of clones was decreased (Figure 2E) and that after upregulation of UBE2S, the number of clones was increased (Figure 2F). Furthermore, wound healing assays and transwell assays were used to explore the potential role of UBE2S in the regulation of HCC cell migration. We found that knockdown of UBE2S decreased the wound healing rate (Figure 3A), while overexpression of UBE2S increased the wound healing rate (Figure 3B). Similarly, the transwell assays showed that downregulation of UBE2S caused fewer cells to pass through the membranes (siUBE2S) than did those of the control group (sicontrol) (Figure 3C), while overexpression of UBE2S (pUBE2S) increased the number of invasive cells compared with the control group (MOCK) (Figure 3D). In addition, knockdown of UBE2S decreased HCC cell invasion, while overexpression of UBE2S increased HCC cell invasion (Figure 3E). Western blotting (Figure 3F,G) and immunofluorescence assays (Figure 3H,I) demonstrated that downregulation of UBE2S upregulated the expression levels of E‐cadherin (the epithelial cell marker) and downregulated the expression levels of vimentin and N‐cadherin (the mesenchymal cell markers), while overexpression of UBE2S had the opposite results. In addition, UBE2S also regulated the EMT transcription factor snail (Figure 3F,G). Taken together, these results showed that in HCC, UBE2S could promote the malignant properties by increasing cell proliferation, migration, and invasion in vitro.

### 3.3 Downregulation of UBE2S inhibited HCC growth and metastasis in vivo

**FIGURE 4:**
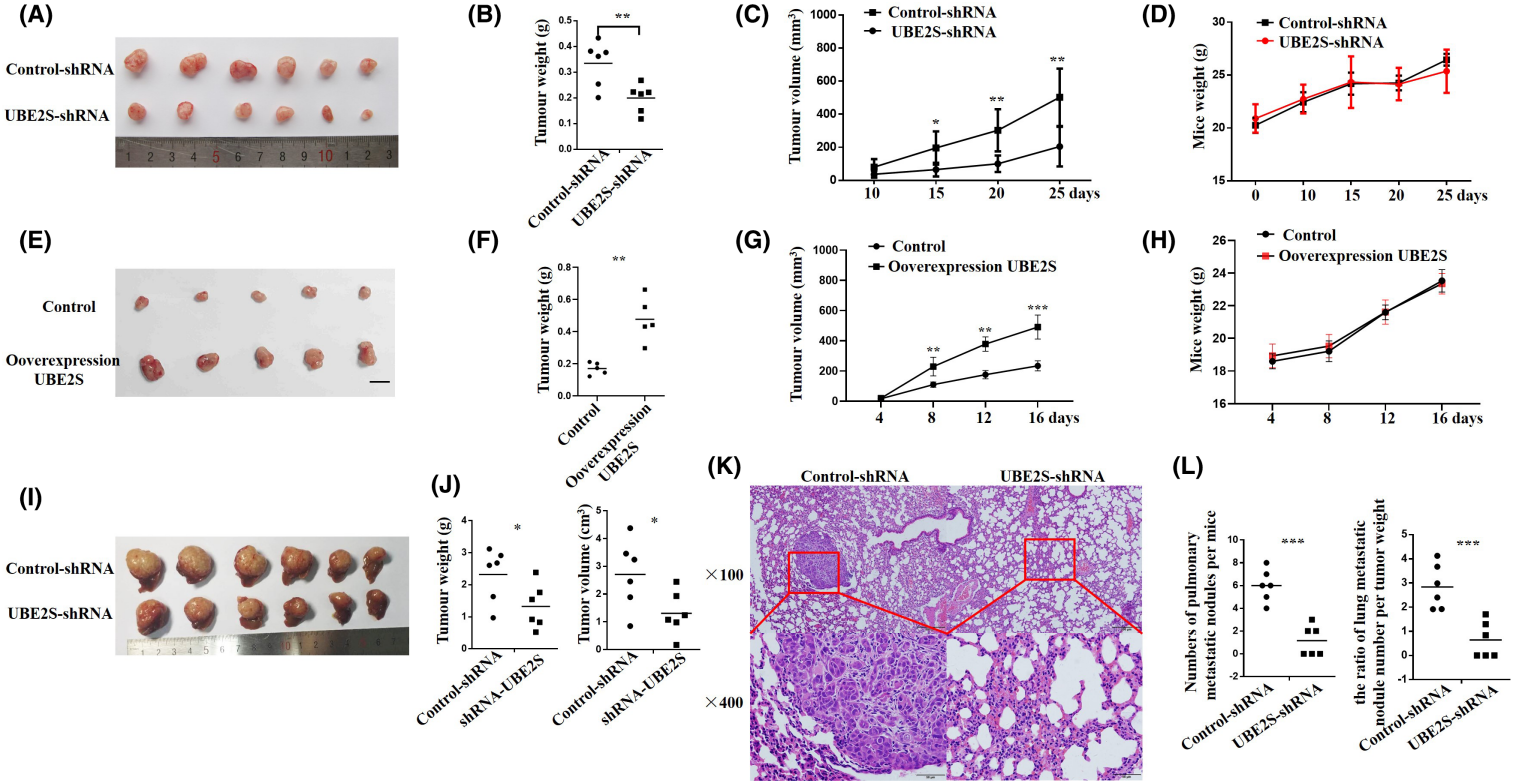
UBE2S promotes HCC growth and metastasis in vivo. (A) Tumors from the UBE2S‐shRNA and control‐shRNA groups in subcutaneous xenograft tumor models were removed 25 days after tumor inoculation, and photos of the tumors were taken. (B) The weights of tumors from the UBE2S‐shRNA and control‐shRNA groups in subcutaneous xenograft tumor models were measured. (C) The volumes of tumors in the UBE2S‐shRNA and control‐shRNA groups in subcutaneous xenograft tumor models were measured on different days after tumor inoculation. (D) The weights of mice from the UBE2S‐shRNA and control‐shRNA groups in subcutaneous xenograft tumor models were measured. (E) Tumors from the UBE2S‐overexpressing Bel‐7402 cells and control groups in subcutaneous xenograft tumor models were removed 16 days after tumor inoculation, and photos of the tumors were taken. (F) The weights of tumors from the UBE2S overexpression and control groups in subcutaneous xenograft tumor models were measured. (G) The volumes of tumors in the UBE2S overexpression and control groups in subcutaneous xenograft tumor models were measured on different days after tumor inoculation. (H) The weights of mice from the UBE2S overexpression and control groups in subcutaneous xenograft tumor models were measured. (I) Tumors from the UBE2S‐shRNA and control‐shRNA groups in orthotopic xenograft tumor models were removed 8 weeks after tumor inoculation, and photos of tumors were taken. (J) The tumors were excised from livers in orthotopic xenograft tumor models, and the weights and volumes of tumors in the UBE2S‐shRNA and control‐shRNA groups were measured. (K) Representative images of H&E staining of pulmonary metastatic nodules in the orthotopic HCC model (left panel). Quantitation of the pulmonary metastatic nodules is shown in the right panel. (L) Quantitation of the ratio of lung metastatic nodule number per tumor weight (g) for each mouse is shown. The data are presented as the mean ± SD. *p < 0.05; **p < 0.01; ***p < 0.001.

To evaluate the effect of UBE2S on HCC progression in vivo, HCCLM3 cells with stable knockdown of UBE2S expression (named UBE2S‐shRNA HCCLM3) and Bel‐7402 cells with stable overexpression of UBE2S (named UBE2S‐ overexpression Bel‐7402) were established via lentivirus transfection. Then, we used UBE2S‐shRNA HCCLM3 cells and control‐shRNA HCCLM3 cells to establish subcutaneous xenograft tumor models and orthotopic xenograft tumor models. The results showed that downregulation of UBE2S significantly inhibited tumor size (Figure 4A). The tumor sizes and weights of the UBE2S‐shRNA group were markedly decreased (Figure 4B,C). However, there was no obvious distinction in the weights of mice from the UBE2S‐shRNA and control‐shRNA groups in subcutaneous xenograft tumor models (Figure 4D). Moreover, upregulation of UBE2S significantly improved tumor size (Figure 4E). The tumor sizes and weights of the UBE2S overexpression group were markedly increased (Figure 4F,G). There was also no obvious distinction in the weights of mice from the control and UBE2S overexpression groups (Figure 4H).

In addition, in liver orthotopic xenograft tumor models, the results were consistent with those of subcutaneous xenograft tumor models, which indicated that knockdown of UBE2S could inhibit tumor growth in vivo (Figure 4I,J). In addition, H&E staining of lungs confirmed that downregulation of UBE2S in HCC decreased the number of pulmonary metastatic nodules in liver orthotopic xenograft tumor models (Figure 4K). We also determined the ratio of lung metastatic nodule number per tumor weight for each mouse, and there were still significant differences between the two groups, which indicated that UBE2S knockdown reduced metastasis independently of tumor growth (Figure 4L). In summary, the results indicated that UBE2S plays an important role in HCC growth and metastasis in vivo.

### 3.4 Downregulation of UBE2S arrested HCC cells in the G2/M phase by decreasing the degradation of p21

**FIGURE 5:**
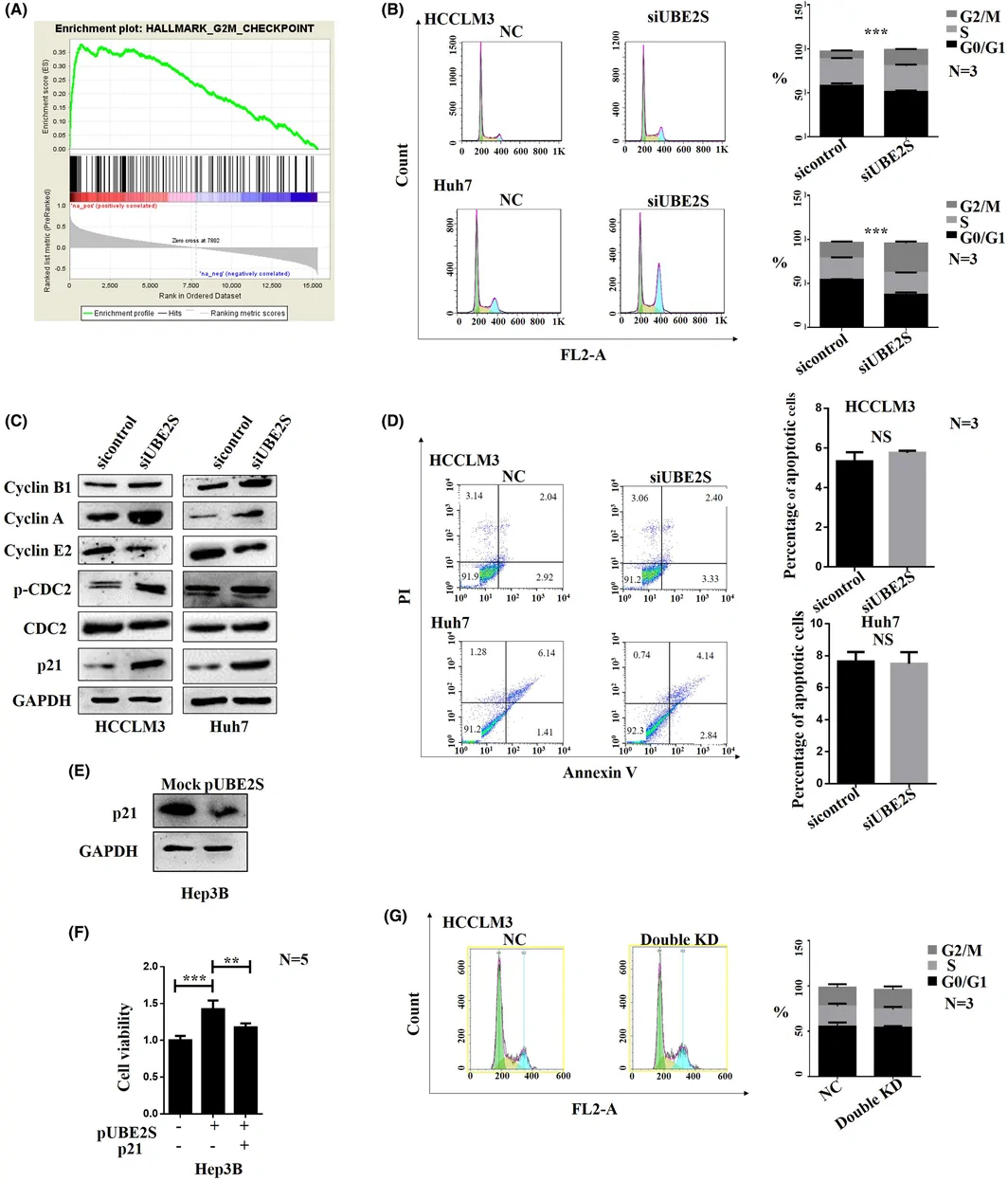
Downregulation of UBE2S arrests HCC cells in the G2/M phase in vitro. (A) Based on TCGA data, gene set enrichment analysis (GSEA) was launched to explore which signaling pathways were upregulated in HCC cases with UBE2S overexpression. (B) Cell cycle analysis of HCC cells after knockdown of UBE2S was measured through flow cytometry. Quantitation of the percentage of the cells in G1/G0, S and G2/M phases is shown in the right panel. (C) The expression levels of G2/M‐arrest‐related proteins were assayed by western blotting. (D) Apoptosis in cells with downregulated expression of UBE2S was measured by flow cytometry. Quantitation of the percentage of apoptotic cells is shown in the right panel. (E) p21 was downregulated by UBE2S overexpression. (F) Overexpression of p21 reversed the effects of UBE2S upregulation on HCC proliferation. (G) Cell cycle analysis of HCCLM3 cells after knockdown of UBE2S and P21 (double KD) was measured through flow cytometry. Quantitation of the percentage of the cells in G1/G0, S and G2/M phases is shown in the right panel. The data are presented as the mean ± SD. NS: no significance; *p < 0.05; **p < 0.01; ***p < 0.001.

To explore the molecular mechanism of downregulation of UBE2S‐mediated inhibition of HCC growth, gene set enrichment analysis (GSEA) was conducted based on TCGA data. The results showed that the G2/M checkpoint signaling pathway was upregulated in HCC patients with high expression of UBE2S mRNA (Figure 5A). Then, the cell cycle of HCCLM3 and HUH7 cell lines after knockdown of UBE2S was detected by flow cytometry. The percentage of cells in G2/M was increased compared with that of control cells (Figure 5B). In addition, we further measured the G2/M‐arrest‐related proteins in HCCLM3 and HUH7 cell lines after UBE2S knockdown. The results indicated that cyclin A, cyclin B1, and p‐CDC2 were upregulated and cyclin E2 was downregulated in the HCCLM3 and HUH7 cell lines after UBE2S knockdown (Figure 5C). These results indicated that downregulation of UBE2S arrested HCC cells in the G2/M phase. More importantly, apoptosis assays were conducted, and there was no difference between the siUBE2S group and the sicontrol group, which indicated that downregulation of UBE2S suppressed HCC cell growth by arresting the cell cycle rather than inducing cell apoptosis (Figure 5D).

**FIGURE 6:**
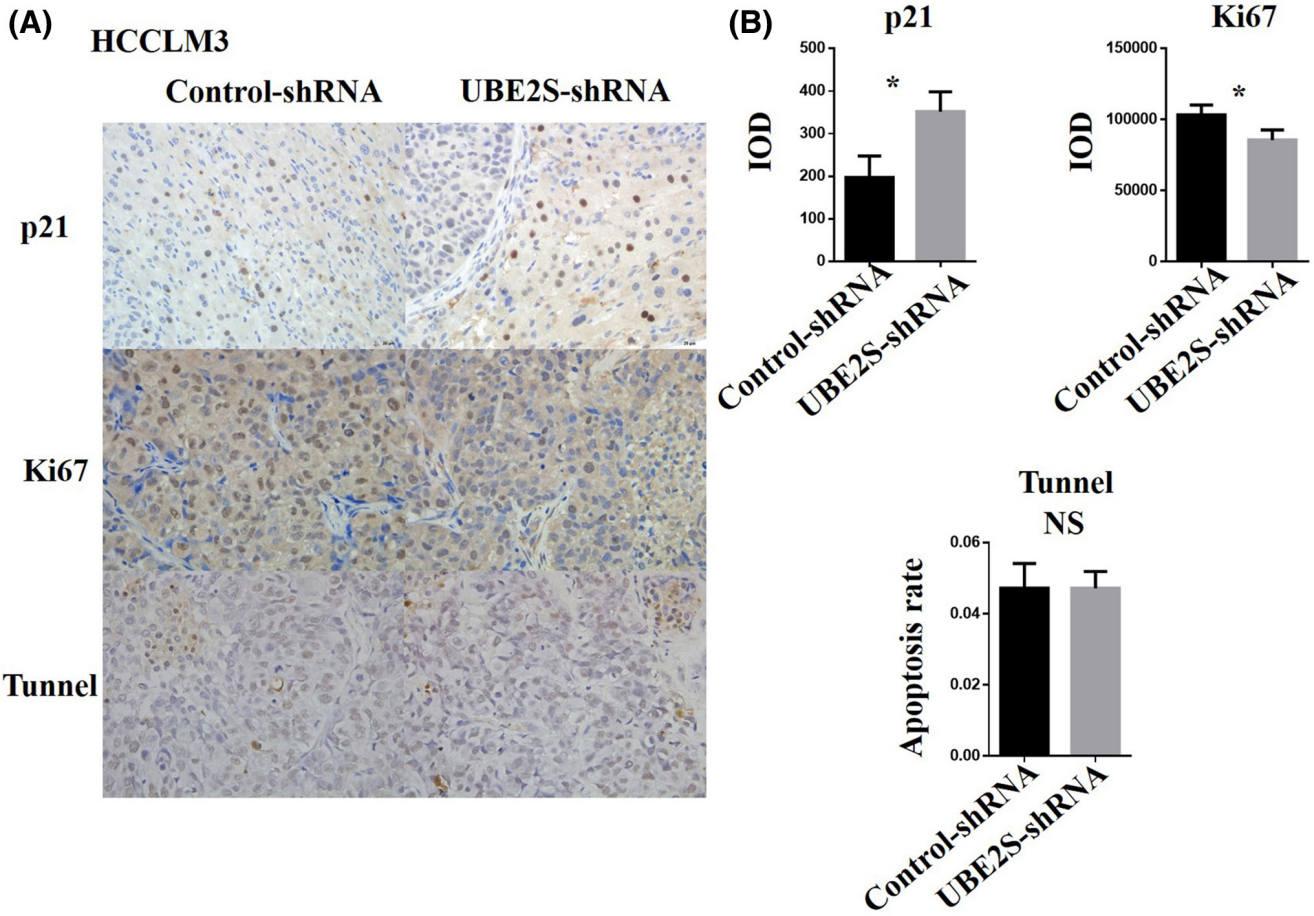
IHC experiments for p21 protein, Ki67‐positive cells and TUNEL staining were carried out in HCC tumor tissues from a subcutaneous xenograft model. (A) Representative images of p21 protein, Ki67‐positive cells, and TUNEL staining are illustrated in the left panel. (B) Quantitation of the integral optical density (IOD) is shown. The data are presented as the mean ± SD. *p < 0.05; **p < 0.01; ***p < 0.001.

Many studies have shown that the p21 protein is a regulator of the cell cycle at the G2/M checkpoint.^29^, ^30^, ^31^ In addition, knockdown of UBE2S can arrest OSCC cells in the G2/M phase by decreasing the degradation of p21.^16^ Inspired by the abovementioned findings, we detected the expression levels of p21 protein. The results indicated that knockdown of UBE2S increased p21 protein levels (Figure 5C). In addition, p21 was downregulated by UBE2S overexpression in Hep3B cells (Figure 5E). To examine the role of p21 in UBE2S‐mediated cell proliferation, p21 was upregulated after overexpression of UBE2S in Hep3B cells. The results incated that overexpression of p21 reversed the upregulation effects of UBE2S on HCC cell proliferation (Figure 5F). Moreover, downregulation of p21 reversed the effect of arresting HCC cells in the G2/M phase after inhibition of UBE2S in HCCLM3 cells (Figure 5G). In subcutaneous xenograft tumor models, the p21 protein levels in the UBE2S knockdown group (UBE2S‐shRNA) were significantly increased compared with those in the control‐shRNA group, while there were fewer Ki67‐positive cells in the UBE2S knockdown group than in the control‐shRNA group (Figure 6A,B). However, TUNEL staining revealed no differences between the two groups (Figure 6A,B). All these results demonstrated that downregulation of UBE2S arrested HCC cells in the G2/M phase to inhibit cell proliferation by decreasing the degradation of p21.

### 3.5 UBE2S promoted HCC growth and metastasis by inactivating not only VHL/HIF‐1α signaling but also the VHL/JAK2/STAT3 signaling pathway

**FIGURE 7:**
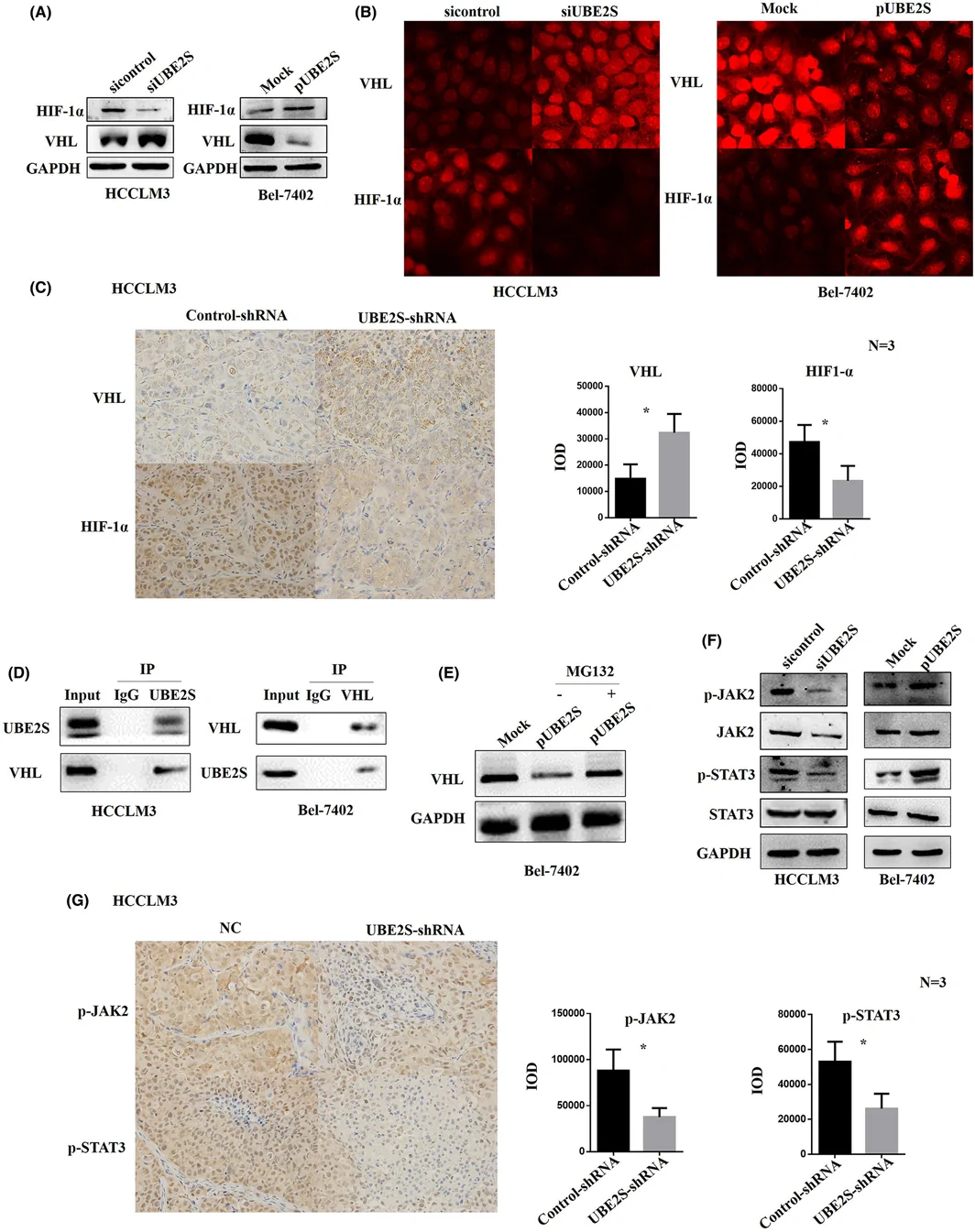
UBE2S promotes HCC growth and metastasis by inactivating not only VHL/HIF‐1α signaling but also the JAK2/STAT3 pathway. (A, B) The expression of VHL and HIF‐1α in HCC cells after upregulation and downregulation of UBE2S was determined by western blotting and immunofluorescence. (C) Representative images of VHL and HIF‐1α protein tested by IHC in liver tissues from a subcutaneous xenograft model. Quantitation of the IOD is shown in the right panel. (D) A coimmunoprecipitation assay was performed to analyse the direct binding between UBE2S and VHL in HCC cells in the presence of 10 mM MG132. (E) The levels of VHL in BEL‐7402 cells after transfection with UBE2S plasmid in the presence or absence of MG132. (F) The expression of STAT3, p‐STAT3, JAK2, and p‐JAK2 was determined by western blotting after upregulation and downregulation of UBE2S in HCC cells. (G) Representative images of p‐STAT3 and p‐JAK2 protein tested by IHC from a subcutaneous xenograft model. Quantitation of the IOD is shown in the right panel. The data are presented as the mean ± SD. *p < 0.05; **p < 0.01; ***p < 0.001.

Previous studies have shown that UBE2S can regulate tumor development through the VHL/HIF‐1α signaling pathway.^21^, ^22^ In addition, UBE2S can target VHL and stabilize HIF‐α in the mouse liver.^15^ However, the key mechanisms of UBE2S in promoting HCC growth and metastasis still needs in‐depth study. Based on these findings, we first verify whether UBE2S promotes HCC growth and metastasis by inactivating VHL/HIF‐1α signaling and their moderating relationship. We detected the protein levels of VHL and HIF‐1α in HCC cells with downregulated or overexpressed UBE2S by western blotting. The results indicated that the expression of VHL protein in HCCLM3 cells was increased significantly after downregulation of UBE2S, and the expression level of HIF‐1α protein was decreased significantly (Figure 7A). In contrast, after the overexpression of UBE2S in Bel‐7402 cells, the expression of VHL in Bel‐7402 cells was significantly inhibited, and the level of HIF‐1α protein was increased (Figure 7A). We then further measured the expression of VHL and HIF‐1α by immunofluorescence. The results were similar to the western blotting results (Figure 7B). Moreover, the protein expression levels of VHL and HIF‐1α in tumor tissues collected from xenograft tumor models were detected by IHC, and the results were similar to those from western blotting (Figure 7C).

In addition, we tried to explore the direct interaction between UBE2S and VHL in HCC cells by co‐IP. First, HCCLM3 cells were transfected with the UBE2S plasmid and then incubated for 12 h in the presence of 10 mM MG132, a proteasome inhibitor, and the cell lysates were immunoprecipitated with anti‐UBE2S antibodies. Then, western blotting was used to detect the expression level of VHL. The results showed that VHL was contained in the cell lysates immunoprecipitated with anti‐UBE2S antibodies (Figure 7D). Similarly, UBE2S was also detected in cell lysates immunoprecipitated with anti‐VHL antibodies in Bel‐7402 cells (Figure 7D). We also examined the levels of VHL in Bel‐7402 cells after transfection with the UBE2S plasmid and incubation for 12 h in the presence or absence of 10 mM MG132. This result showed that VHL was decreased in a manner dependent on the expression level of UBE2S in the absence of MG132 and this decrease was eliminated in the presence of MG132 (Figure 7E). In addition, Jung et al. and Lim et al. also showed that UBE2S targeted VHL for ubiquitin‐mediated proteolysis to degrade VHL (15, 21). Taken together, UBE2S modulates VHL/HIF‐1α signal transduction by binding to VHL and degrading VHL.

It has been reported that VHL can regulate the transduction of JAK/STAT signaling.^32^, ^33^ Therefore, we speculated that in HCC cells, UBE2S may regulate the transduction of JAK/STAT signaling by directly interacting with VHL. To verify the role of UBE2S in the regulation of the JAK/STAT signaling pathway in HCC, we detected the protein levels of JAK2, p‐JAK2, STAT3, and p‐STAT3. The results indicated that the protein levels of p‐JAK2 and p‐STAT3 in HCC cells were decreased after downregulating the expression of UBE2S (Figure 7F). In contrast, the upregulated expression of UBE2S in Bel‐7402 cells increased the protein levels of p‐JAK2 and p‐STAT3 (Figure 7F). Furthermore, we detected the protein levels of p‐JAK2 and p‐STAT3 in tumor tissues collected from HCC xenograft tumor models by IHC. The results showed that the expression of p‐JAK2 and p‐STAT3 proteins in the UBE2S‐shRNA group was significantly lower than that in the control‐shRNA group (Figure 7G).

In conclusion, UBE2S may regulate HCC growth and metastasis by inactivating not only VHL/HIF‐1α signaling but also the VHL/JAK2/STAT3 pathway.

### 3.6 Downregulation or upregulation of VHL reversed the effect of UBE2S on cell proliferation and migration

**FIGURE 8:**
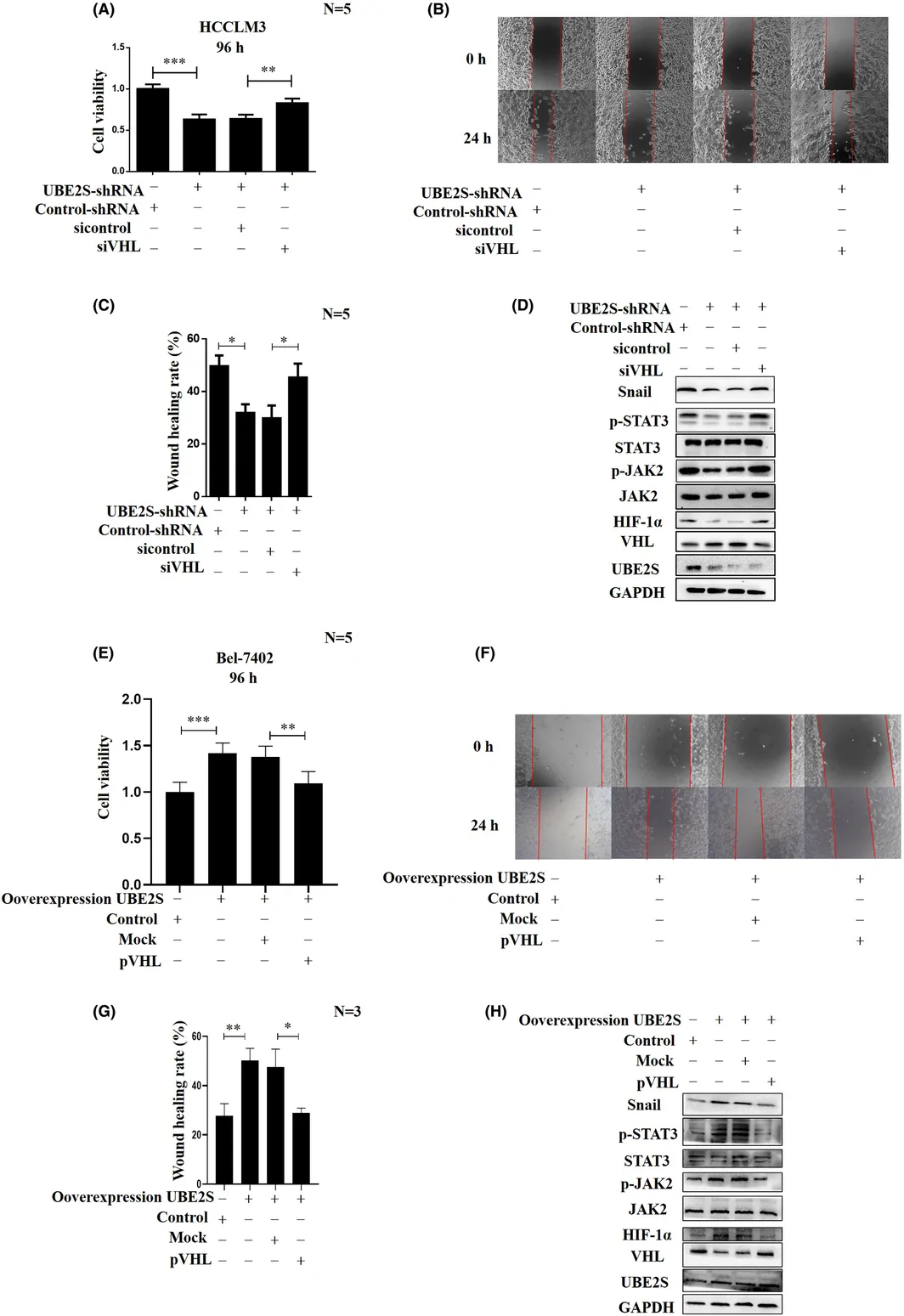
Downregulation or upregulation of VHL reversed the effect of UBE2S on cell proliferation and migration. (A, B) After the expression of VHL was downregulated in HCCLM3 cells with stable knockdown of UBE2S, MTT, and wound healing assays were performed to measure the influence of VHL on the proliferation and migration abilities. (C) Quantitation of the cell wound healing rate in HCCLM3 cells is shown. (D) After downregulating the expression levels of VHL in HCCLM3 cells with stable knockdown of UBE2S, western blot analysis of UBE2S, VHL, Snail, HIF1α, STAT3, p‐STAT3, JAK2, and p‐JAK2 expression was carried out. (E, F) After the expression of VHL was upregulated in UBE2S‐overexpressing Bel‐7402 cells, MTT, and wound healing assays were performed to measure the influence of VHL on the proliferation and migration abilities. (G) Quantitation of the cell wound healing rate in Bel‐7402 cells is shown. (H) After upregulating the expression levels of VHL in UBE2S‐overexpressing Bel‐7402 cells, western blot analysis of UBE2S, VHL, Snail, HIF1α, STAT3, p‐STAT3, JAK2, and p‐JAK2 expression was carried out. The data are presented as the mean ± SD. *p < 0.05; **p < 0.01; ***p < 0.001.

Because downregulation of UBE2S increases VHL protein levels, we questioned whether the effect of downregulation of UBE2S in HCC is attributable to the enhancement in VHL stability. We cotransfected a lentivirus to generate stable UBE2S knockdown and siVHL in HCCLM3 cells and examined cell proliferation and migration. The influence of VHL on cell proliferation at 96 h was detected by MTT assays (Figure 8A). In Figure 8B,C, the migration abilities were also determined. Indeed, siVHL reversed the inhibition of cell proliferation and migration mediated by UBE2S knockdown (Figure 8A–C). We found that knockdown of VHL reversed the inhibition of cell proliferation and migration caused by the downregulation of UBE2S in HCCLM3 cells (Figure 8A–C). We further measured the expression levels of Snail, HIF‐1α, JAK2, p‐JAK2, STAT3, and p‐STAT3. The results indicated that knockdown of VHL in UBE2S‐shRNA HCCLM3 cells increased the expression levels of Snail, HIF‐1α, JAK2, p‐JAK2, and p‐STAT3 (Figure 8D).

In addition, we also checked whether overexpression of VHL could reverse the accelerated cell proliferation, migration, and invasion mediated by UBE2S overexpression. UBE2S‐overexpressing Bel‐7402 cells were cotransfected with pVHL, and cell proliferation and migration were examined. pVHL reversed the enhancement of cell proliferation and migration mediated by UBE2S overexpression in Bel‐7402 cells (Figure 8E–G). We further measured the protein levels of Snail, HIF‐1α, JAK2, p‐JAK2, STAT3, and p‐STAT3. The results showed that VHL overexpression in UBE2S cells reduced the protein levels of Snail, HIF‐1α, JAK2, p‐JAK2, and p‐STAT3 (Figure 8H).

The data suggested that VHL plays a key role in UBE2S‐mediated HCC development, in which UBE2S could bind VHL and promote VHL degradation to activate VHL/HIF‐1α signaling and the VHL/JAK2/STAT3 pathway.

### 3.7 Knockdown of UBE2S increases the sensitivity of HCC cells to sorafenib

**FIGURE 9:**
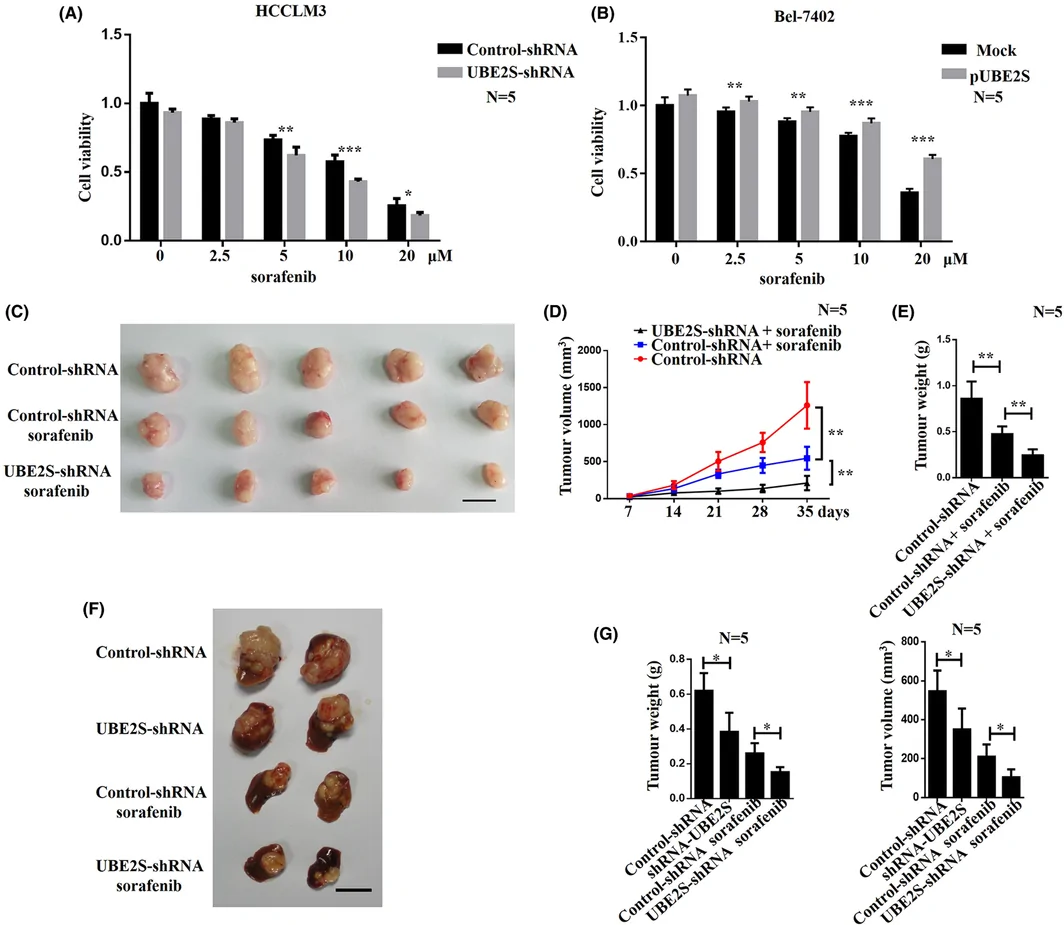
Knockdown of UBE2S increases the sensitivity of HCC cells to sorafenib. (A) HCCLM3 cells with UBE2S knockdown (UBE2S‐shRNA) were treated with sorafenib, and the viability of the cells was detected. HCC cells with normal UBE2S expression (control‐shRNA) treated with sorafenib were used as controls. (B) Bel‐7402 cells overexpressing UBE2S (pUBE2S) were treated with sorafenib, and the viability of the cells was detected. HCC cells with empty vector (Mock) treated with sorafenib were used as controls. (C) HCCLM3 cells (2 × 106) with normal or downregulated expression of UBE2S were subcutaneously injected into nude mice, and the mice were treated with sorafenib (50 mg/kg/d) for 35 days (named the control‐shRNA+sorafenib group and UBE2S‐shRNA+sorafenib group, respectively), and the tumors were obtained. Scale bar, 1 cm. (D, E) The volumes and weights of the tumors were assessed. (F) In liver orthotopic xenograft tumor models, the mice were treated with sorafenib, and typical images of the tumors were obtained. Scale bar, 1 cm. (G) The volumes and weights of the tumors were assessed. The data are presented as the mean ±  SD. *p < 0.05; **p < 0.01; ***p < 0.001.

To measure the biological significance of UBE2S in the sensitivity of HCC cells to sorafenib, the viability of HCC cells treated with sorafenib was detected. We found that after downregulation of UBE2S in HCCLM3 cells, the cell death induced by sorafenib was markedly increased (Figure 9A). The half maximal inhibitory concentrations (IC_50_) of sorafenib in HCCLM3‐control‐shRNA cells and HCCLM3‐UBE2S‐shRNA cells were 10.57 μM and 8.68 μM, respectively. In addition, upregulation of UBE2S enhanced sorafenib resistance in Bel‐7402 cells (Figure 9B). The IC50 of sorafenib was 15.87 and 24.57 μM before and after overexpression of UBE2S in Bel‐7402 cells, respectively. To further explore the role of UBE2S in sorafenib sensitivity in vivo, we established subcutaneous xenograft models with UBE2S‐shRNA HCCLM3 and their negative control cells, and then the nude mice were treated with sorafenib daily by intraperitoneal injection. The results indicated that tumors in the UBE2S‐shRNA group treated with sorafenib were smaller than those in their negative control group treated with sorafenib (Figure 9C–E). In addition, in liver orthotopic xenograft tumor models, the results also indicated that tumors in the UBE2S‐shRNA group treated with sorafenib were smaller than those in their negative control group treated with sorafenib, suggesting that knockdown of UBE2S made HCC cells more sensitive to sorafenib in vivo (Figure 9F,G).

## 4 DISCUSSION

Ubiquitination has significant effects in cellular homeostasis and involves three enzymes: ubiquitin‐activating enzyme (E1), ubiquitin‐conjugating enzyme (E2), and ubiquitin ligase (E3). UBE2S is an E2 enzyme, which can elongate the ubiquitin chains on target proteins.^34^ Many studies have confirmed that UBE2S plays a central role in diverse biological processes, such as the cell cycle, cell growth, signal transduction, and transcription.^35^, ^36^ As in tumors, previous studies had showed that UBE2S is overexpressed in renal, breast, oesophageal, cervical, HCC, and brain glioma cancer, which was related to tumor and tissue specificity.^17^, ^19^, ^22^, ^23^, ^37^, ^38^ In addition, high expression of UBE2S indicates a more unfavourable prognosis in oesophageal squamous cell carcinoma and brain glioma.^37^, ^38^ Considering the situation in HCC, Pan et al. found that UBE2S is associated with poor outcomes in HCC and enhances the ubiquitination of p53 to promote HCC development.^17^ In addition, UBE2S interacting with TRIM28 in the nucleus can promote HCC development through accelerating the cell cycle by ubiquitination of p27.^19^ However, the role of UBE2S in HCC cell proliferation, migration, and the molecular mechanism in vivo, has not yet been verified. More importantly, the underlying mechanism by which UBE2S overexpression regulates HCC progression is far from fully understood.

In the present study, our data indicated that HCC patients with high expression level of UBE2S had shorter disease‐free and overall survival via Kaplan–Meier analyses (*p* = 0.014 and 0.036, respectively), which indicated that UBE2S in HCC is associated with poor prognosis. Overexpression of UBE2S in HCC cells markedly promoted cell proliferation, migration, and invasion, while knockdown of UBE2S had the opposite effects in vitro. Our study was similar to Pan's report and strongly indicated that UBE2S is a tumor oncogene in HCC and may be a novel potential therapeutic marker for HCC. In addition, we further measured the role of UBE2S in HCC cell proliferation, migration and invasion, as well as the underlying mechanism in vivo, and first found that downregulation of UBE2S could induce cell cycle arrest at G2/M phase in HCC cells. In our data, cell cycle analysis of HCC cells indicated that downregulation of UBE2S suppressed HCC cell proliferation by arresting HCC cells in the G2/M phase rather than inducing cell apoptosis. In addition, the p21 protein levels were significantly increased after downregulation of UBE2S in HCC cells, and overexpression of p21 reversed the upregulation effects of UBE2S on HCC cell proliferation. More importantly, the experiment showed that downregulation of p21 reverses the effect of arresting HCC cells in the G2/M phase after inhibition of UBE2S in HCCLM3 cells. Based on these results, our results confirmed that downregulation of UBE2S arrests HCC cells in the G2/M phase, resulting in decreased cell proliferation by decreasing p21 degradation.

Then, we further explored the molecular mechanisms of UBE2S in regulating the occurrence and progression of HCC tumors. Studies have reported that UBE2S targets VHL for degradation to regulate tumor growth and metastasis in various cancers.^15^, ^21^, ^22^ However, the relationship between the UBE2S‐regulated VHL/HIF pathway and HCC progression is still unclear. In our studies, the results indicated that overexpression of UBE2S could significantly decrease the expression levels of VHL, thereby stabilizing HIF‐1α. In contrast, downregulation of UBE2S in HCC cells had the opposite effect. The data also showed that UBE2S directly bound to VHL in HCC cells. More importantly, inhibition of VHL reversed the downregulation of UBE2S‐induced inhibition of cell proliferation and metastasis, as well as the protein levels of HIF‐1α. These results indicated that in HCC cells, UBE2S regulates cell proliferation and metastasis via the VHL/HIF‐1α pathway. In addition, knockdown of VHL in UBE2S‐shRNA HCCLM3 cells increased the protein levels of Snail. Therefore, we think the regulation of Snail may occur through the UBE2S/VHL/HIF‐1α signaling pathways. Although UEB2S was found to modulate the VHL/HIF‐1α pathway in different cell types, we speculated that UBE2S may use a different mechanism to modulate HCC growth and metastasis.

In our data, we found that UBE2S bound to VHL for degradation. Wu et al. reported that VHL targets p‐JAK2 for ubiquitin‐mediated destruction.^32^ In addition, Kanno et al. found that the VHL protein regulates tumorigenicity by inhibiting the JAK/STAT signaling pathway.^33^ The JAK2‐STAT3 pathway regulates the expression and function of a variety of genes that are critical to cell proliferation, angiogenesis, and immune evasion. Recently, targeting the JAK2‐STAT3 signaling pathway has become a hot spot for cancer therapy, and a variety of JAK2‐STAT3 inhibitors have been identified that induce antitumor activity both in vitro and in vivo. Therefore, we speculated that overexpression of VHL, which was caused by downregulation of UBE2S in HCC, may inhibit the JAK/STAT signaling pathway, which could regulate cell proliferation, migration, and invasion. To prove this hypothesis, we measured the protein levels of p‐JAK2 and p‐STAT3 in HCC cells after knockdown or upregulation of UBE2S. The data showed that downregulation of UBE2S inhibited the JAK/STAT signaling pathway in vivo and in vitro. Moreover, when we inhibited the expression of VHL in HCC cells with downregulated UBE2S, the results showed that the inhibition of the JAK/STAT signaling pathway induced by downregulation of UBE2S was reversed. We first demonstrated that UBE2S could regulate the JAK2/STAT3 signaling pathway through VHL degradation. However, the interaction between VHL and p‐JAK2 in HCC cells is still unclear. UBE2S may modulate HCC growth and metastasis via different mechanisms. There may be some connections between different mechanisms. However, more studies are needed.

Recently, UBE2S was shown to mediate chemoresistance in breast cancer and glioblastoma.^19^, ^20^ Knockdown of UBE2S expression increased the sensitivity of breast cancer and glioblastoma cells to etoposide. In HCC, sorafenib is currently an important first‐line drug approved for systemic treatment. However, sorafenib tends to induce adverse side effects and resistance.^39^, ^40^ In addition, the detailed molecular mechanism of acquired sorafenib resistance remains unclear. Interestingly, we found that silencing UBE2S in HCCLM3 cells sensitized the cells to sorafenib. In addition, inhibition of UBE2S in combination with sorafenib synergistically decreased tumor growth in vivo. In conclusion, UBE2S expression could be a potential target for predicting the sorafenib treatment response and therapeutic intervention.

In summary, overexpression of UBE2S in HCC is associated with poor prognosis, and UBE2S may promote HCC cell proliferation and migration via the VHL/HIF‐1α and VHL/JAK2/STAT3 signaling pathways. Moreover, downregulation of UBE2S enhances the effect of sorafenib, which provides a novel marker for sorafenib‐resistant HCC patients, and cotargeting of UBE2S may promote the efficacy of sorafenib.

## AUTHOR CONTRIBUTIONS

**Junyi Wu:** Data curation (equal); formal analysis (equal); methodology (equal); software (equal); writing – original draft (equal); writing – review and editing (equal). **Xiangjie Xu:** Formal analysis (equal); methodology (equal). **Shasha Wu:** Data curation (equal); investigation (equal). **Weiwei Shi:** Data curation (equal); formal analysis (equal). **Guang Zhang:** Data curation (equal); methodology (equal); project administration (equal). **Yin Cao:** Data curation (equal). **zhongxia wang:** Conceptualization (equal); data curation (equal); funding acquisition (equal). **junhua wu:** Conceptualization (equal); formal analysis (equal); writing – review and editing (equal). **chunping jiang:** Conceptualization (equal); formal analysis (equal); funding acquisition (equal); investigation (equal); project administration (equal).

## FUNDING INFORMATION

This work was supported by the National Natural Science Foundation of China (81972888, 82272819); the Research Project of Jinan Microecological Biomedicine Shandong Laboratory (JNL202219B, JNL202204A, JNL‐2023017D); Shandong Provincial Laboratory Project (SYS202202); the Primary Research & Development Plan of Jiangsu Province (BE2022840); the Startup Fund for scientific research, Fujian Medical University (2018QH1112); and the Open Project of Chinese Materia Medica First‐Class Discipline of Nanjing University of Chinese Medicine (No. 2020YLXK007).

## CONFLICT OF INTEREST STATEMENT

The authors declare that they have no conflicts of interest.

## ETHICAL APPROVAL STATEMENT

Human liver cancer tissue specimens were obtained following the guidelines approved by the ethics committee of the Affiliated Drum Tower Hospital of Nanjing University Medical School, and all patients gave written informed consent used for research purposes. All animal protocols were approved by the Animal Care Committee of Nanjing University in accordance with the guidelines of the Institutional Animal Care and Use Committee.

## Supporting information


Table S1.

## Data Availability

The datasets used in our study can be obtained from the corresponding authors according to reasonable request.
