# Supplementary material for: UBE2S promotes malignant properties via VHL/HIF‐1α and VHL/JAK2/STAT3 signaling pathways and decreases sensitivity to sorafenib in hepatocellular carcinoma
Source: Cancer Med. 2023 Aug 10;12(17):18078–97. doi: 10.1002/cam4.6431 (PMC10523983; doi:10.1002/cam4.6431)
Supplement: Supplementary file 1 — Table S1. [file CAM4-12-18078-s001.docx]

| **Clinicopathological features** |  |  | **UBE2S** | | ***P* value** |
| --- | --- | --- | --- | --- | --- |
|  |  |  | **Low *n* (%) High *n* (%)** | |  |
| Sex  Age  HBsAg  AFP (ng/mL)  Cirrhosis  Tumor number  Tumor size (cm)  vascular invasion  TNM stage | M  F  ＞60  ≤60  +  -  ＞20  ≤20  +  -  ＞1  1  ＞5  ≤5  +  -  II-IV  I | 87  16  39  64  77  26  66  37  84  19  25  78  62  41  36  67  73  30 | 42(48.3%)  5(31.3%)  17(43.6%)  30(46.7%)  34(44.2%)  13(50%)  27(40.9%)  20(54.1%)  39(46.4%)  8(42.1%)  11(44%)  36(46.2%)  23(37.1%)  24(58.5%)  14(38.9%)  33(49.3%)  28(38.4%)  19(63.3%) | 45(51.7%)  11（68.7%）  22(56.4%)  34(53.3%)  43(55.8%)  13(50%)  39(59.1%)  17(45.9%)  45(53.6%)  11(57.9%)  14(56%)  42(53.8%)  39(62.9%)  17(41.5%)  22(61.1%)  34(50.7%)  45(61.6%)  11(36.7%) | 0.209  0.839  0.605  0.199  0.733  0.851  **0.032***  0.314  **0.021*** |
